# Identification of molecular heterogeneity in SNX27–retromer-mediated endosome-to-plasma-membrane recycling

**DOI:** 10.1242/jcs.156299

**Published:** 2014-11-15

**Authors:** Ian J. McGough, Florian Steinberg, Matthew Gallon, Ayaka Yatsu, Norihiko Ohbayashi, Kate J. Heesom, Mitsunori Fukuda, Peter J. Cullen

**Affiliations:** 1 The Henry Wellcome Integrated Signaling Laboratories, School of Biochemistry, Medical Sciences Building, University of Bristol, Bristol BS8 1TD, UK; 2 Department of Developmental Biology and Neurosciences, Graduate School of Life Sciences, Tohoku University, Sendai 980-8578, Japan; 3 Proteomics Facility, School of Biochemistry, Medical Sciences Building, University of Bristol, Bristol BS8 1TD, UK

**Keywords:** SNX27, VARP, VPS35, Retromer, Sorting nexin

## Abstract

Retromer is a protein assembly that orchestrates the sorting of transmembrane cargo proteins into endosome-to-Golgi and endosome-to-plasma-membrane transport pathways. Here, we have employed quantitative proteomics to define the interactome of human VPS35, the core retromer component. This has identified a number of new interacting proteins, including ankyrin-repeat domain 50 (ANKRD50), seriologically defined colon cancer antigen 3 (SDCCAG3) and VPS9-ankyrin-repeat protein (VARP, also known as ANKRD27). Depletion of these proteins resulted in trafficking defects of retromer-dependent cargo, but differential and cargo-specific effects suggested a surprising degree of functional heterogeneity in retromer-mediated endosome-to-plasma-membrane sorting. Extending this, suppression of the retromer-associated WASH complex did not uniformly affect retromer cargo, thereby confirming cargo-specific functions for retromer-interacting proteins. Further analysis of the retromer–VARP interaction identified a role for retromer in endosome-to-melanosome transport. Suppression of VPS35 led to mistrafficking of the melanogenic enzymes, tyrosinase and tryrosine-related protein 1 (Tyrp1), establishing that retromer acts in concert with VARP in this trafficking pathway. Overall, these data reveal hidden complexities in retromer-mediated sorting and open up new directions in our molecular understanding of this essential sorting complex.

## INTRODUCTION

On arriving in the endosomal network, internalized transmembrane protein cargos have two principal fates – either they are sorted from the limiting membrane of the endosome into intraluminal vesicles for delivery to the lysosome for degradation or, alternatively, they are exported from the endosome for recycling to the plasma membrane or to the trans-Golgi network (TGN) (Huotari and Helenius, 2011). Although much has been learned about how cargos are selected and sorted into the degradative pathway, how these sorting events are regulated during retrieval of cargos into the various export pathways remains poorly understood.

Pioneering studies in yeast led to the identification of a pentameric protein complex termed ‘retromer’ that was essential for the recycling of the Vps10 sorting receptor from endosomes to the TGN (Seaman et al., 1997; Seaman et al., 1998). In yeast, retromer is composed of two subcomplexes – a membrane remodeling heterodimer of the SNX-BAR [sorting nexins (SNX) that possess Bin/Amphiphysin/Rvs (BAR) domains] proteins Vps5 and Vps17, and a heterotrimer, classically termed the cargo-selective complex (CSC), that is composed of Vps26, Vps29 and Vps35 and has been shown to recognize cargo including Vps10 (Seaman et al., 1998). Retromer in yeast therefore serves as a coat complex for co-ordinating cargo selection and cargo enrichment with membrane remodeling to generate cargo-enriched transport carriers for endosome-to-TGN transport (Burd and Cullen, 2014).

The CSC is an ancient protein assembly that evolved before the last common eukaryotic ancestor and has been highly conserved throughout eukaryotic evolution (Koumandou et al., 2011). Unsurprisingly, in higher metazoans, the role of retromer has expanded, with the CSC residing as the core component of three sorting complexes – the SNX-BAR–retromer, the SNX3–retromer and the SNX27–retromer (Carlton et al., 2004; Wassmer et al., 2009; Harterink et al., 2011; Temkin et al., 2011; Zhang et al., 2011; Steinberg et al., 2013). These complexes have distinct roles not only in endosome-to-TGN recycling but also endosome-to-plasma-membrane transport, and they act to sort a wide array of functionally distinct cargos (Cullen and Korswagen, 2012). The retromer CSC has therefore emerged as a master conductor in the orchestration of multiple endosomal sorting events. Moreover, deregulation of retromer function is emerging in a number of human diseases, including Alzheimer disease and Parkinson disease (Muhammad et al., 2008; Vilariño-Güell et al., 2011; Wen et al., 2011; Zimprich et al., 2011), and as a target during host–pathogen interactions (Kingston et al., 2011; Finsel et al., 2013; Lipovsky et al., 2013; McDonough et al., 2013).

The diversification of the roles of retromer has raised the fundamental question of how the common CSC is able to sort such a wide array of cargo proteins into distinct endosome-to-TGN and endosome-to-plasma-membrane transport pathways (Burd and Cullen, 2014). Potentially providing insight into this underlying heterogeneity is an emerging view that, besides its role in cargo selection, the retromer CSC is a recruitment hub for the endosomal association of a number of accessory proteins that aid retromer-mediated sorting. Included among these accessory factors are TBC1D5, a potential Rab GTPase-activating protein for the late-endosome-associated Rab7 (Seaman et al., 2009), and the Wiskott-Aldrich syndrome protein and SCAR homolog (WASH) complex, a multi-protein complex that, by regulating Arp2/3, promotes the formation of branched actin filaments on the endosomal network (Derivery et al., 2009; Gomez and Billadeau, 2009).

To further explore the role of the CSC as an endosomal scaffold and provide additional insight into the functional heterogeneity of retromer, we here describe the employment of stable isotope labeling with amino acids in cell culture (SILAC)-based labeling and high-resolution mass spectrometry to quantify the interactome of human VPS35. In addition to established interactors, including all components of the WASH complex, this has identified a number of new retromer accessory proteins that we have functionally validated in the context of retromer-mediated endosome-to-plasma-membrane recycling. These include ANKRD50, a multiple ankyrin-repeat domain containing protein with no described function; SDCCAG3, an endosome-associated protein implicated in cytokinesis (Hagemann et al., 2013), and VARP (also known as ANKRD27), an endosome-associated effector for Rab32 and Rab38 that possesses guanine-nucleotide exchange activity against Rab21 (Zhang et al., 2006) and traps the R-SNARE VAMP7 in a closed fusogenically inactive conformation and thereby regulates late endosome fusion with the lysosome (Schäfer et al., 2012). Through comparative analysis of four functionally distinct SNX27–retromer cargos – the glucose transporter GLUT1 (also known as SLC2A1), the monocarboxylate transporter MCT1, the TNF-receptor super-family member TRAILR1 (also known as TNFRSF10A) and the adhesion receptor CD97 – we reveal underlying mechanistic heterogeneity in retromer-mediated endosome-to-plasma-membrane transport and provide a molecular framework to aid the further analysis of retromer function. Finally, in describing a requirement for retromer in VARP-mediated endosome transport of the melanogenic enzymes tyrosinase and tryrosine-related protein 1 (Tyrp1) during melanosome biogenesis (Tamura et al., 2009), we identify a new role for retromer in the biogenesis of this lysosome-related organelle.

## RESULTS

### Quantitative proteomic analysis of the VPS35 interactome

**Fig. 1.**
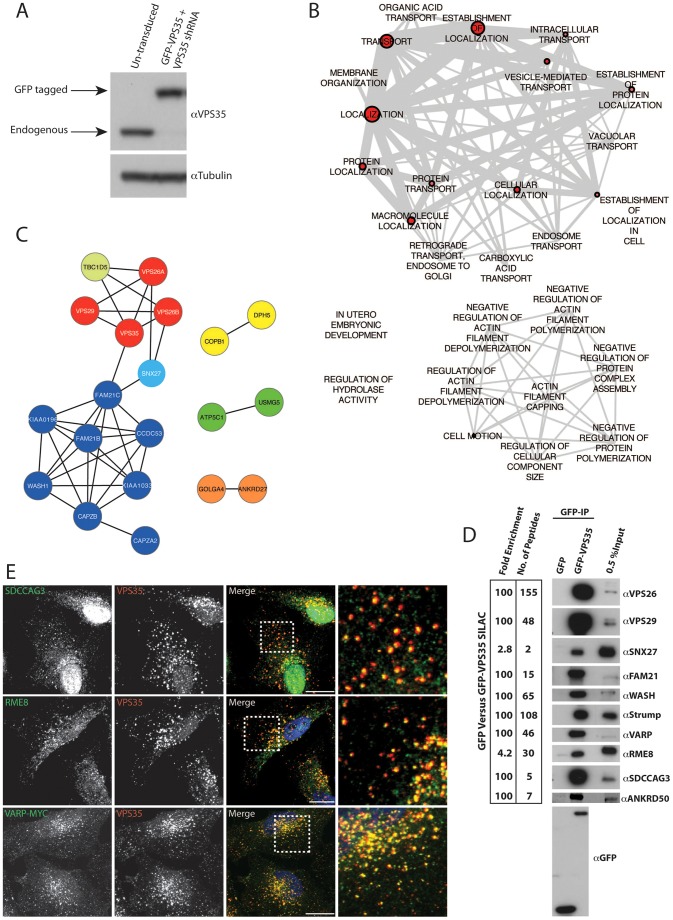
**Identification of the VPS35 interactome using a quantitative SILAC proteomic approach reveals novel endosomally localized retromer interactors.** (A) Lysates from control RPE1 cells and RPE1 cells with stable suppression of endogenous VPS35 (using an shRNA lentivirus targeting the 3′ UTR), prior to the stable re-expression of GFP–VPS35, were immunoblotted with anti-VPS35 and anti-tubulin antibodies. (B) Gene Ontology annotations revealed a preponderance of proteins involved in ‘establishment of localization’, ‘localization’ and ‘transport’ in the VPS35 interactome. DAVID was used to assign Gene Ontology annotations to proteins identified in the VPS35 SILAC proteomics with a >2.5-fold enrichment and with a minimum of two peptides. The larger the red node, the greater the number of proteins classified in that category; the thicker the edge between nodes, the greater the overlap of proteins within those classifications. (C) The majority of known retromer interactors were found in the VPS35 interactome. Network analysis of VPS35 interactome components identified in the SILAC proteomics was performed using the STRING database. Colours represent protein–protein interactions or protein complexes known to associate. (D) Novel interactions were confirmed by western blotting. Cell extracts derived from RPE1 cells lentivirally transduced with GFP or GFP–VPS35 were subjected to a GFP-nanotrap (GFP-IP) and subsequently analyzed for binding to the indicated proteins. The number of peptides and fold enrichment of the indicated proteins in the VPS35 SILAC proteomics are also indicated. (E) VARP, SDCCAG3 and RME-8 colocalize with VPS35. HeLa cells transiently transfected with VARP–Myc and untransfected HeLa cells were fixed and stained with antibodies raised against Myc, RME-8 or SDCCAG3 and co-stained with an antibody against endogenous VPS35 (red). Boxed areas are shown at higher magnification to the right. Scale bars: 10 µm.

To gain new and unbiased insight into the mechanistic basis of retromer-mediated endosomal sorting, we sought to obtain a quantitative high-resolution interactome of human VPS35, the core component of the retromer CSC (Seaman et al., 1998). To this end, we first generated an RPE1 cell line in which the expression of endogenous VPS35 was stably suppressed through lentiviral short hairpin (sh)RNA-mediated targeting of the 3′UTR. Into this cell line, we stably re-expressed GFP-tagged VPS35 at close to endogenous levels (Fig. 1A). The resultant cell line was cultured in heavy SILAC medium alongside an RPE1 cell line stably expressing GFP that was cultured in light SILAC medium. Following cellular lysis, GFP was precipitated using the highly efficient GFP-trap method (Trinkle-Mulcahy et al., 2008). Precipitates were combined, separated by SDS-PAGE and quantified by liquid chromatography-tandem mass spectrometry (LC-MS/MS) after in-gel tryptic digestion. From 2530 quantified proteins (supplementary material Table S1), 63 proteins were considered to comprise the VPS35 interactome after filtering of the data by two criteria – protein quantification from two or more peptides and an enrichment of >2.5-fold over control. Gene ontology analysis revealed that the majority of these interactors had roles in ‘establishment of localization’, ‘localization’ and ‘transport’ (Fig. 1B). Network analysis (Fig. 1C) confirmed the presence of the known VPS35-interacting partners VPS26A and VPS26B, VPS29, TBC1D5 (Harbour et al., 2010), SNX27 (Steinberg et al., 2013) and all components of the actin polymerizing WASH complex (Derivery et al., 2009; Gomez and Billadeau, 2009; Harbour et al., 2010; Harbour et al., 2012; Jia et al., 2012; Hao et al., 2013). The lack of enrichment of the SNX-BAR components of the SNX-BAR–retromer is entirely consistent with the apparent low affinity of this interaction [the interaction of VPS29 with SNX1 has a K_d_ of >150 µM (Swarbrick et al., 2011)].

Besides these established binding partners, a number of new interactors were identified (supplementary material Table S2). Of these, we selected the top three proteins, each with high levels of enrichment and numerous quantified peptides, for further validation and functional analysis. These proteins were VARP (>100-fold enriched, 46 quantified peptides), ANKRD50 (>100-fold enriched, seven quantified peptides) and SDCCAG3 (>100-fold enriched, five quantified peptides). In addition, we further characterized the role of RME-8 (also known as DNAJC13), a protein previously implicated in retromer-mediated sorting (Popoff et al., 2009; Shi et al., 2009; Freeman et al., 2014), and the WASH complex component FAM21, which, through direct binding to VPS35, mediates the association of WASH with the CSC (Derivery et al., 2009; Gomez and Billadeau, 2009; Harbour et al., 2010; Harbour et al., 2012; Jia et al., 2012). Western blot analysis of immunoprecipitated GFP–VPS35 versus GFP confirmed the enrichment of each protein (Fig. 1D), and immunofluorescence analysis established that endogenous SDCCAG3 and RME-8, and a transiently expressed myc-tagged VARP, were associated with retromer-decorated endosomes (Fig. 1E).

### RNAi-mediated suppression of ANKRD50 phenocopies loss of SNX27–retromer function

To establish the functional role of the new retromer-associated proteins, we individually suppressed the expression of all three interactors, as well as that of RME-8 and FAM21, and examined the endosome-to-plasma-membrane recycling of a variety of SNX27–retromer-dependent cargos, initially focusing on the glucose transporter GLUT1 (Steinberg et al., 2013). At steady state, GLUT1 is localized at the plasma membrane from where it undergoes continuous rounds of endocytosis and PDZ-motif-dependent endosome-to-plasma-membrane recycling (Wieman et al., 2009), the latter being mediated by the SNX27–retromer (Steinberg et al., 2013). RNA interference (RNAi)-mediated suppression of SNX27 or the retromer CSC component VPS35 leads to a pronounced decrease in the amount of GLUT1 at the cell surface and a corresponding decrease in whole-cell levels, as the transporter undergoes missorting and enhanced degradation in the lysosome (Steinberg et al., 2013). Hence, we established procedures for analyzing GLUT1 trafficking in HeLa cells by confocal microscopy and through biochemical analysis of whole-cell levels and the specific expression of this transporter at the cell surface.

**Fig. 2.**
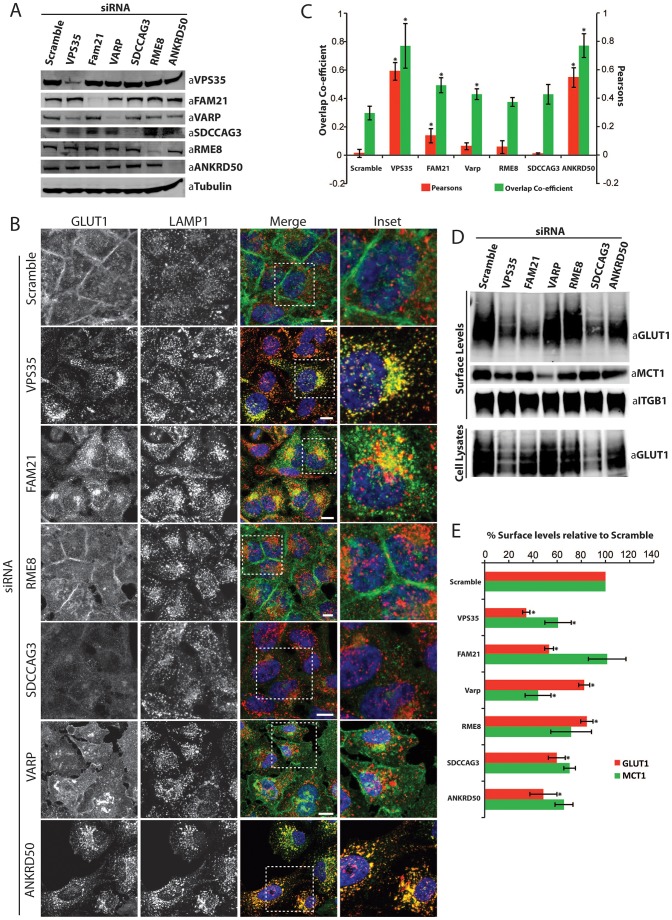
**Suppression of the novel interactors identified in the VPS35 SILAC proteomics screen affects retromer-mediated endosome-to-plasma-membrane transport.** (A) Western blot analysis of lysates derived from HeLa cells that were transfected with siRNAs against the indicated targets. Tubulin is shown as a loading control. (B,C) VPS35, ANKRD50 and FAM21 suppression leads to an increase in the lysosomal accumulation of GLUT1. Immunofluorescent staining of endogenous GLUT1 and the lysosomal marker LAMP1 in HeLa cells deficient for VPS35, FAM21, RME-8, SDCCAG3, VARP or ANKRD50 (B). Boxed areas are shown at higher magnification to the right. Scale bars: 10 µm. (C) The data show the mean±s.e.m. (150 cells acquired in three independent experiments, *n* = 3); *P<0.05 (unpaired Student's *t*-test). (D,E) Surface levels of MCT1 and GLUT1 are decreased upon suppression of certain retromer interactors. HeLa cells were transfected with the indicated siRNAs and the surface abundance of GLUT1 and MCT1 was determined by quantitative western blotting (D). The abundance of GLUT1 in total cell lysates is also shown. (E) Graphical representation of the loss of GLUT1 and MCT1 from the surface of HeLa cells transfected with the indicated siRNAs. Data show the mean±s.e.m. [three (MCT1) and six (GLUT1) independent experiments]; *P<0.05 (unpaired Student's *t*-test).

**Fig. 3.**
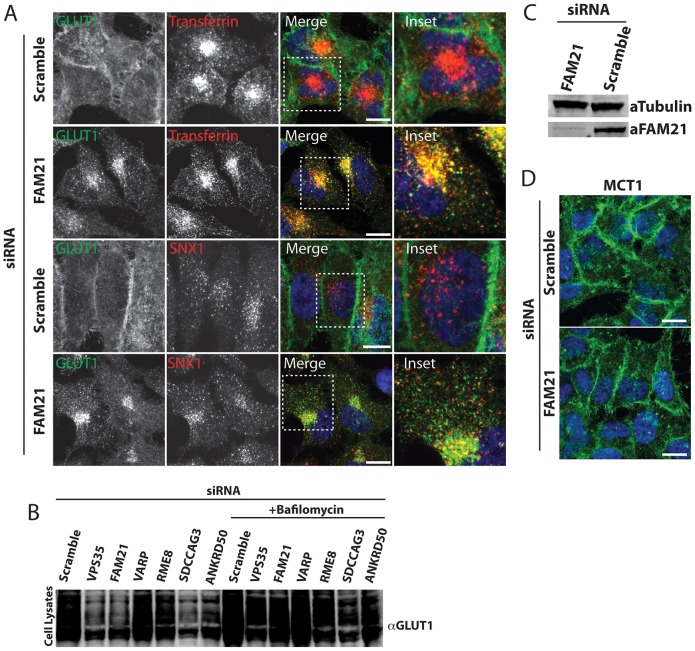
**FAM21 suppression leads to an intracellular accumulation of GLUT1 in a SNX1- and transferrin-positive compartment.** (A) Immunofluorescent staining of endogenous GLUT1 and SNX1, or GLUT1 and dsRed–transferrin after 1 h uptake in FAM21-deficient HeLa cells. Boxed areas are shown at higher magnification to the right. Scale bars: 10 µm. (B) HeLa cells were transfected with the indicated siRNAs and, 48 h later, were treated with either DMSO or 0.1 µM bafilomycin in DMSO for a further 12 h prior to analysis of GLUT1 levels in total-cell lysates. (C) Western blot analysis of lysates derived from HeLa cells that were transfected with siRNAs against the indicated targets. Tubulin is shown as a loading control. (D) Immunofluorescent staining of endogenous MCT1 in control and FAM21-deficient HeLa cells. Scale bars: 10 µm.

In HeLa cells, RNAi-mediated suppression of VPS35, FAM21, VARP, SDCCAG3, RME-8 and ANKRD50 was highly efficient (Fig. 2A). In control cells, GLUT1 was mostly localized to the plasma membrane, indicating efficient recycling (Fig. 2B). Like suppression of VPS35, suppression of ANKRD50 resulted in a loss of GLUT1 expression at the cell surface, which corresponded to an increase in GLUT1 localization to a LAMP1-labeled lysosomal compartment (Fig. 2B,C). Moreover, suppression of ANKRD50 led to a decrease in surface expression of GLUT1, as determined through a surface biotinylation protocol, and a decrease in the whole-cell level of the transporter (Fig. 2D,E). Entirely consistent with a missorting of internalized GLUT1 into the lysosomal-degradative pathway, the observed decrease in the whole-cell level of the GLUT1 was reversed by treating ANKRD50-suppressed cells with bafilomycin to inhibit lysosomal degradation (Fig. 3B). Finally, and to be discussed in more detail later, suppression of ANKRD50 also phenocopied a loss of SNX27–retromer function in the endosome-to-plasma-membrane sorting of MCT1, CD97 and TRAILR1. Taken together, these data identify a role for ANKRD50 in the endosome-to-plasma-membrane sorting of multiple cargos and provide evidence that, mechanistically, its role in this process is linked with the SNX27–retromer sorting complex.

### Analysis of other retromer accessory proteins reveals heterogeneity in retromer-mediated GLUT1 transport

Consistent with the previously reported requirement for WASH1 in the regulation of GLUT1 recycling in T-lymphocytes (Piotrowski et al., 2013), suppression of the WASH complex component FAM21 also decreased GLUT1 surface expression and reduced-whole cell levels of GLUT1 (Fig. 2B–E). However, FAM21 suppression did not completely phenocopy retromer or ANKRD50 suppression, as only a small portion of internalized GLUT1 localized to the LAMP1-labeled lysosome (Fig. 2B,C); the majority appeared to be trapped in a SNX1- and transferrin-labeled endocytic recycling compartment (ERC) (Fig. 3A). Interestingly, this apparent heterogeneity in the requirement of specific retromer accessory proteins for efficient SNX27–retromer-mediated GLUT1 trafficking extended to SDCCAG3, RME-8 and VARP.

**Fig. 4.**
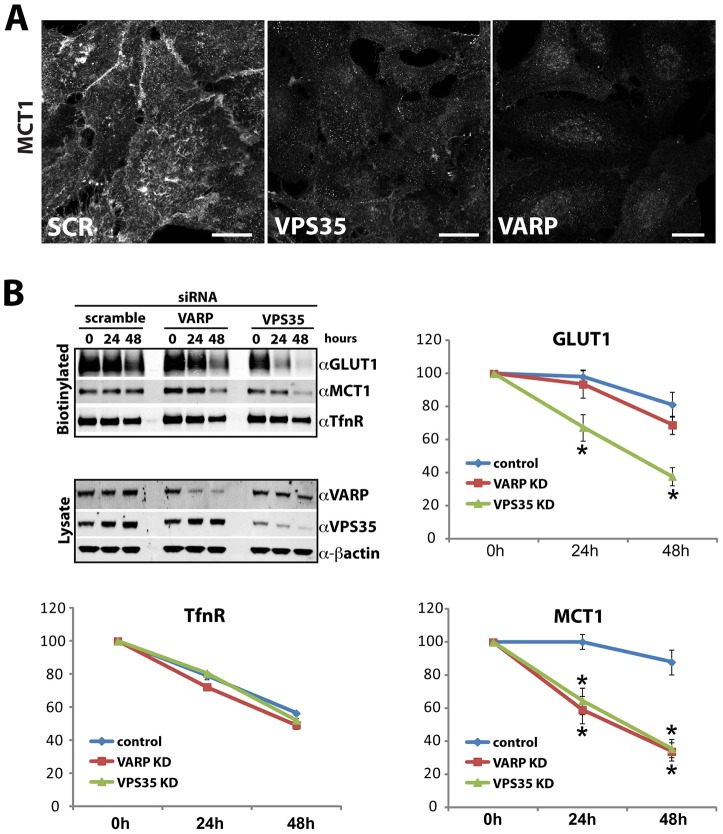
**VARP suppression affects endosome-to-plasma-membrane transport of the SNX27–retromer cargos MCT1 and CD97.** (A) VARP suppression leads to a decrease in MCT1 immunofluorescent signal. HeLa cells transfected with siRNA against VARP or VPS35 were fixed and stained for MCT1. Scale bars: 10 µm. (B) Degradation assays of biotin-labeled SNX27–retromer cargo. HeLa cells were transfected with siRNA against VARP and VPS35 and were surface biotinylated at 24 h post transfection, before the effects of suppression became fully evident. Biotinylated proteins were captured from lysates with streptavidin beads at the indicated time points after biotinylation and subjected to quantitative western blotting on an Odyssey scanner. β-actin is shown as a loading control. VARP suppression results in increased degradation kinetics of MCT1. The data represent the mean±s.e.m. (ten independent experiments); *P<0.05 (unpaired Student's *t*-test).

Suppression of SDCCAG3 led to a decrease in GLUT1 surface expression and a reduction in whole-cell levels, but this did not correlate with an obvious accumulation of GLUT1 in lysosomes (Fig. 2B–E) or the ERC (data not shown). Furthermore, the reduction in the whole-cell level of GLUT1 could only be partially rescued by blocking lysosomal degradation (Fig. 3B), consistent with a complex phenotype that might include a level of GLUT1 transcriptional control. Suppression of neither RME-8 nor VARP generated a robust GLUT1 phenotype when analyzed by immunofluorescent or biochemical procedures (Fig. 2B–E), although subsequent quantification revealed a mild but significant disruption of GLUT1 trafficking (Fig. 2C,E). To provide a further independent analysis of the role of VARP in GLUT1 sorting, we quantified the kinetics of GLUT1 degradation following VARP suppression. Again, VARP suppression failed to phenocopy the clear increase in GLUT1 degradation kinetics observed upon retromer suppression (Fig. 4B). Overall, although VARP associates with retromer and is localized to retromer-decorated endosomes, it is not functionally required for the SNX27–retromer-mediated endosome-to-plasma-membrane sorting of GLUT1.

### For other SNX27–retromer cargos, VARP is required for their endosome-to-plasma-membrane transport

**Fig. 5.**
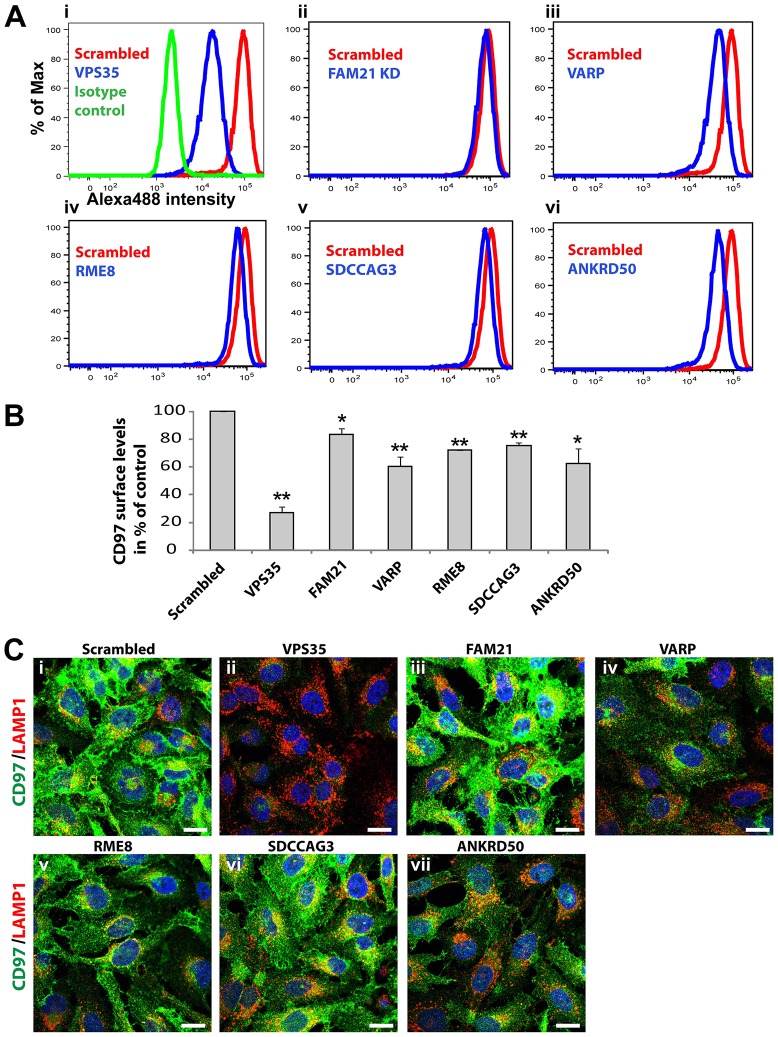
**CD97 surface levels are perturbed by the suppression of VPS35 interactors.** (A,B) Flow cytometric analysis of surface-resident CD97 in cells deficient for FAM21, RME-8, SDCCAG3, ANKRD50, VARP or VPS35. Data in B show the mean±s.e.m.; *P<0.025; **P<0.01 (unpaired Student's *t*-test). (C) HeLa cells transfected with siRNA against the indicated targets were fixed and stained to examine the distribution of endogenous CD97 and its colocalization with LAMP1-decorated late endosomes and lysosomes. Scale bars: 10 µm.

**Fig. 6.**
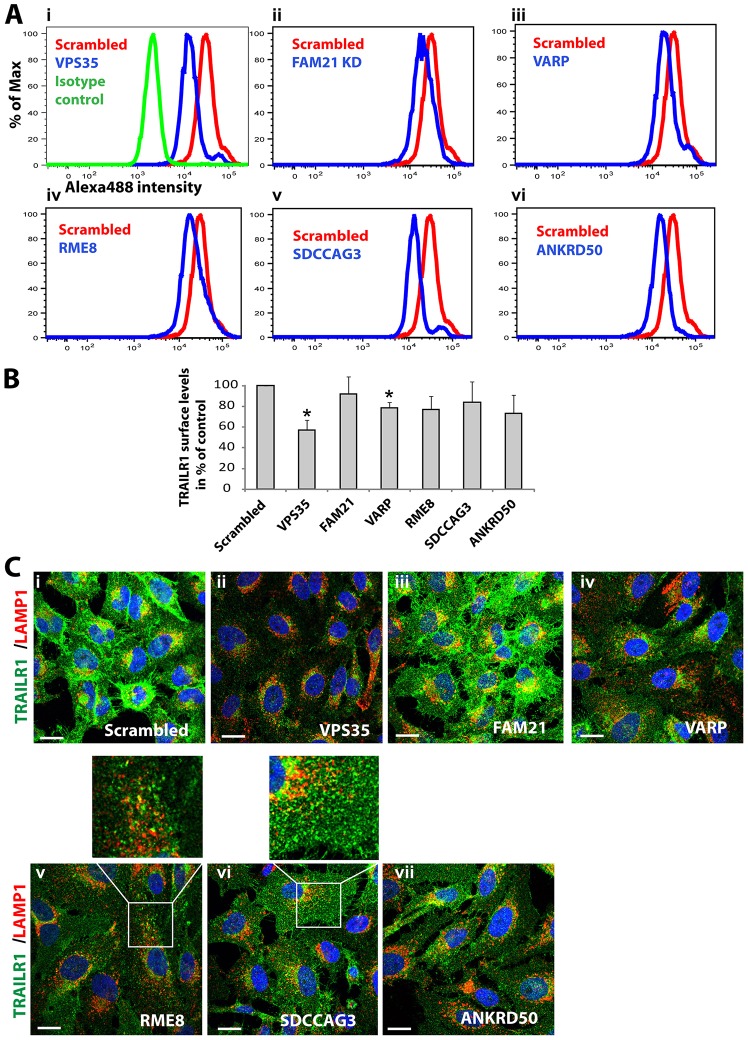
**TRAILR1 surface levels are perturbed by the suppression of VPS35 interactors.** (A,B) Flow cytometric analysis of surface-resident TRAILR1 in cells deficient for FAM21, RME-8, SDCCAG3, ANKRD50, VARP and VPS35. Data in B show the mean±s.e.m.; *P<0.025 (unpaired Student's *t*-test). (C) HeLa cells transfected with siRNA against the indicated targets were fixed and stained to examine the distribution of endogenous TRAILR1 and its colocalization with LAMP1-decorated late endosomes and lysosomes. Boxed areas are shown at higher magnification above the main image. Scale bars: 10 µm.

In contrast to the subtle effects on GLUT1, subsequent examination of the additional SNX27–retromer cargos MCT1, CD97 and TRAILR1 revealed a functional link between retromer and VARP. Suppression of VARP and, as published previously, VPS35 (Steinberg et al., 2013) led to a pronounced reduction in cell-surface and total levels of MCT1, as defined by western blot analysis (Fig. 2D,E) and immunofluorescence (Fig. 4A). Biochemical analysis of the degradation kinetics of MCT1 established that the reduced abundance of this transporter was due to an enhanced degradation rate in VARP-suppressed cells (Fig. 4B). This was specific for MCT1, as GLUT1 and the transferrin receptor displayed normal degradation rates in parallel assays (Fig. 4B). Further analysis of VARP-suppressed cells, employing flow cytometry and imaging-based assays, also revealed greatly reduced cell-surface and total levels of CD97 (Fig. 5Aiii,B,Civ) and TRAILR1 (Fig. 6Aiii,B,Civ), and TRAILR1 uptake assays using an antibody against an exofacial epitope of this receptor also revealed an increase in the lysosomal accumulation of internalized TRAILR1 (supplementary material Fig. S1Aiv). All of these data are indicative of endosomal missorting leading to an enhanced degradation for these SNX27–retromer cargos under VARP-suppressed conditions.

### Further evidence of heterogeneity within the SNX27–retromer pathway is provided through analysis of additional retromer accessory proteins

To further probe the molecular heterogeneity in SNX27–retromer-mediated endosome-to-plasma-membrane sorting, we performed parallel assays under conditions where additional retromer accessory proteins were suppressed. It was notable that disruption of the WASH complex through FAM21 depletion had no or only minor effects on MCT1, CD97 and TRAILR1 trafficking (Fig. 2D,E; Fig. 3C,D; Fig. 5Aii,Ciii; Fig. 6B,Ciii; supplementary material Fig. S1Aiii). Thus, of those SNX27–retromer cargos examined in the present study, only GLUT1 appears to require association with FAM21 and the actin polymerizing WASH complex for optimal endosomal sorting and transport.

For RME-8 and SDCCAG3, RNAi-mediated suppression produced minor effects on CD97 surface levels (Fig. 5Aiv,v) and the surface expression of TRAILR1 (Fig. 6Aiv,v). For TRAILR1, this correlated with an increase in intracellular accumulation (Fig. 6Cv,vi). Similarly, an increased lysosomal accumulation was observed using the TRAILR1 uptake assays (supplementary material Fig. S1Av,vi).

Finally, and as briefly discussed previously, suppression of ANKRD50 expression generally phenocopied a loss of SNX27–retromer. From flow cytometric analysis, suppression of ANKRD50 led to an ∼35% decrease in the surface levels of CD97 (Fig. 5Avi), alongside a similar decrease in the surface expression of TRAILR1 (Fig. 6Avi). For both CD97 and TRAILR1, ANRD50 suppression led to their increased intracellular accumulation (Fig. 5Cvii; Fig. 6Cvii), which we confirmed for TRAILR1 through uptake assays (supplementary material Fig. S1Avii).

### VARP interacts with retromer through a region in its N-terminus

**Fig. 7.**
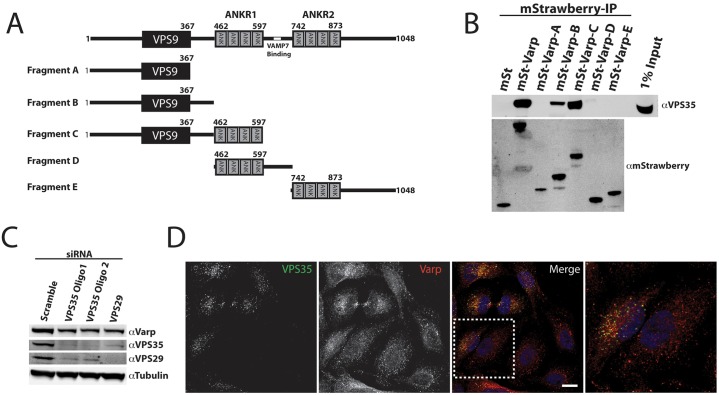
**Retromer binds to the N-terminal region of VARP.** (A) Schematic representation of the VARP constructs. (B) VPS35 interacts with the N-terminal region of VARP. HEK293 cells were transiently transfected with the indicated VARP constructs. At 48 hours post transfection, cells were lysed and the mStrawberry (mSt)-tagged proteins were precipitated using the RFP-trap method, and the immunoprecipitates were subsequently immunoblotted with anti-VPS35 and anti-RFP antibodies. (C) Knockdown of retromer components causes a decrease in VARP protein levels. Lysates from HeLa cells transfected with siRNA targeting VPS29 and two different siRNA oligos targeting VSP35 were immunoblotted with anti-VPS35, anti-VPS29 and anti-VARP antibodies. Tubulin is shown as a loading control. (D) VPS35 is not required for the endosomal localization of VARP. Two HeLa cell populations were stably transduced with a lentivirus encoding VARP–Myc prior to transfection with either control or VPS35-specific siRNA. After 48 hours, the two populations of cells were mixed and subsequently fixed and stained with anti-VPS35 and anti-Myc antibodies. This allows a direct side-by-side comparison of control versus VPS35-suppressed cells. The boxed area is shown at a higher magnification to the right. Scale bar: 10 µm.

Given the strong functional relationship between retromer and VARP in the endosomal sorting of MCT1, CD97 and TRAILR1, we analyzed the binding of VARP to the retromer complex in more detail. VARP comprises an N-terminal VPS9 domain and two C-terminal ankyrin repeat regions, ANKR1 and ANKR2 (Fig. 7A). Co-immunoprecipitation experiments with various mStrawberry-tagged VARP deletion constructs overexpressed in HEK293 cells were consistent with neither of the isolated ANKR domains being sufficient for retromer binding (Fig. 7B). Robust retromer binding, equivalent to that of full-length VARP, was retained with a construct encoding the N-terminal residues 1–597 spanning the VPS9 domain and ANKR1 (Fragment C in Fig. 7B). Further deletion analysis provided evidence that whereas retromer binding was lost with a VARP deletion mutant expressing residues 1–367 (Fragment A), retromer binding was detected to a mutant expressing residues 1–461 (Fragment B). However, it is important to note that the level of retromer binding was significantly reduced in the 1–461 VARP mutant when compared with that observed for wild-type VARP or the 1–597 VARP deletion mutant (Fig. 7B). This is suggestive of a more complex relationship between the N-terminal, VPS9 and ANKR1 domains in the binding to retromer, which might include the correct folding of each individual domain and their associated regions.

### In HeLa cells, a proportion of VARP requires retromer for its stable expression

We were intrigued to observe that endogenous VARP protein levels were consistently decreased by ∼50% in cells treated with siRNA against VPS35 (Fig. 7C; also evident in Fig. 2A and Fig. 4B). That this was not the result of an off-target effect was established with a second siRNA against VPS35 and further confirmed with an siRNA targeting VPS29 (Fig. 7C); the CSC is a stable heterotrimer in which suppression of an individual subunit leads to overall destabilization and loss of expression of the remaining subunits (Arighi et al., 2004; Seaman, 2004). These data therefore indicate that a proportion of VARP requires association with retromer for its stability. Taken together with the binding of VARP to retromer, their strong colocalization on endosomes and their functional relationship in the sorting of specific SNX27–retromer cargos (Fig. 1C,D), VARP and the retromer CSC appear to form a functional complex on retromer-decorated endosomes.

In light of the evidence that the retromer CSC functions as a scaffold for aiding the endosomal association of various accessory proteins including the WASH complex, we examined whether this was also the case for the endosomal association of VARP. However, and entirely consistent with the ability of VARP to bind to endosome-associated Rab32–GTP and Rab38–GTP (Tamura et al., 2009), RNAi-mediated suppression of VPS35 did not perturb the endosome association of stably transduced myc-tagged VARP (Fig. 7D).

### Retromer is linked to VARP-mediated cargo sorting during melanosome biogenesis

**Fig. 8.**
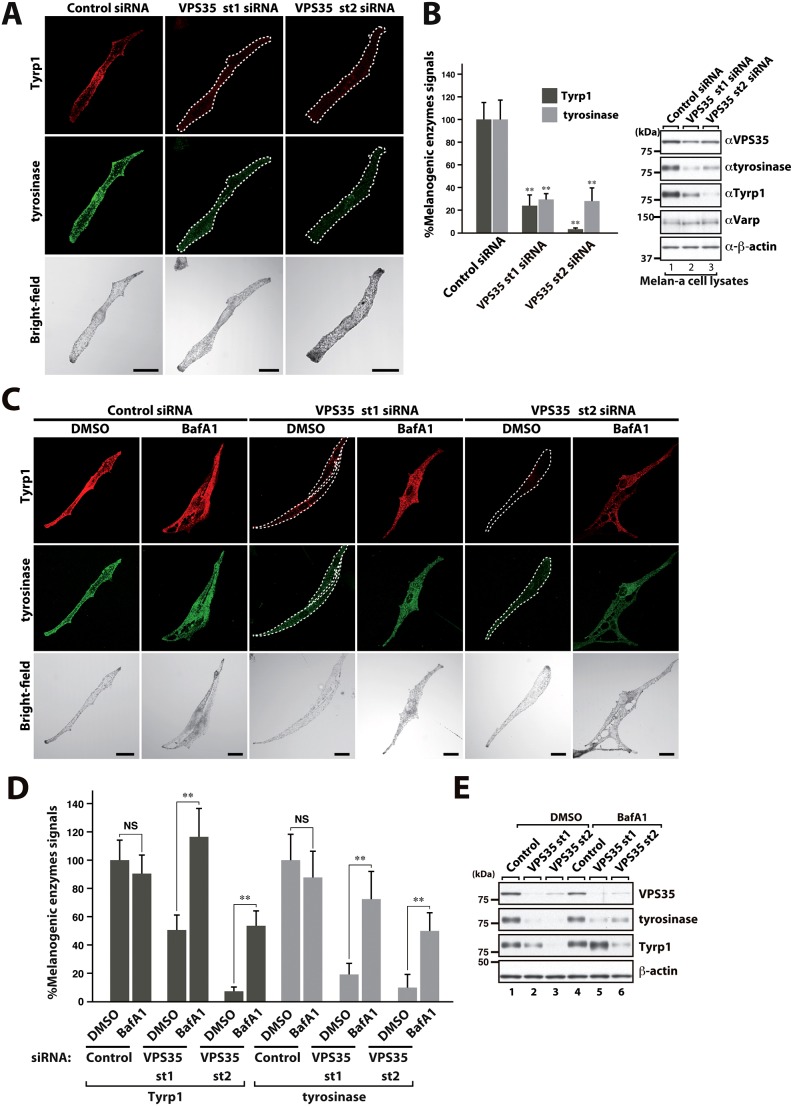
**Retromer plays a role in VARP-mediated trafficking of key melanogenic enzymes.** (A,B) Suppression of VPS35 expression results in a dramatic reduction in the levels of tyrosinase and Tyrp1. Melan-a cells were transfected with control or VPS35-specific siRNA and the cells were fixed and immunostained with anti-Tyrp1 antibody and anti-tyrosinase antibody. Brightfield images show the melanosome distribution in the cells. Scale bars: 20 µm. The level of tyrosinase and Tyrp1 immunofluorescent signals in VPS35-suppressed melanocytes relative to those of controls were quantified and are represented graphically. Cell lysates of melan-a cells treated with either siVPS35 or control siRNA were also subjected to 10% SDS-PAGE followed by immunoblotting with anti-VPS35, anti-Tyrp1, anti-VARP and anti-tyrosinase specific antibodies. β-actin is shown as a loading control. (C–E) Bafilomycin partially rescues the loss of tyrosinase and Tyrp1 caused by VPS35 suppression. The VPS35-knockdown cells were treated with 100 nM bafilomycin A1 (BafA1) for 9 h before fixation. All other procedures were carried out as described for A and B. Scale bars: 20 µm (C). For B,D, data show the mean±s.e.m.; **P<0.01; NS, not significant (Student's *t*-test).

To further validate the functional significance of the observed interaction between retromer and VARP, we turned to analyzing the established role of VARP in melanosome biogenesis. Here, the Rab32/Rab38 and VAMP7 binding activities of VARP, but not its Rab21 guanine nucleotide exchange factor (GEF) activity (Ohbayashi et al., 2012), are required for the endosome sorting and transport of the melanogenic enzymes tyrosinase and Tyrp1 to the maturing melanosome (Tamura et al., 2009). To explore the trafficking of these enzymes, we suppressed retromer in the melanocyte cell line melan-a. Suppression of VPS35 expression, using two independent siRNAs, led to a dramatic reduction in the levels of tyrosinase and Tyrp1, as determined by immunofluorescence and confirmed through western blot analysis (Fig. 8A,B – note that, unlike the situation in HeLa cells, in melan-a cells retromer suppression does not lead to an apparent decrease in VARP expression). Consistent with loss of retromer function leading to the missorting of these enzymes into lysosomes and hence their enhanced degradation, the level of tyrosinase and Tyrp1 were partially restored by treatment with the lysosomal inhibitor bafilomycin A (Fig. 8C–E). Taken together, these data further establish the functional link between retromer and VARP, and suggest that the Rab32/Rab38 and VAMP7 binding activities of VARP act in concert with retromer in the sorting and trafficking of these crucial proteins during melanosome biogenesis.

## DISCUSSION

Here, we have employed quantitative SILAC proteomics to define the interactome of VPS35. Besides detecting most established retromer interactors, which validates our proteomic approach, we identified a number of new interactors, three of which – ANKRD50, VARP and SDCCAG3 – we have characterized in more detail. In addition, we have further clarified the functional significance of the previously identified retromer interactors RME-8 and the WASH component FAM21. For the functional analysis, we focused on the emerging role of SNX27–retromer as a major regulator of endosome-to-plasma-membrane recycling (Temkin et al., 2011; Steinberg et al., 2013). We did so because the robust phenotypes that cargos like GLUT1, MCT1, CD97 and TRAILR1 display upon loss of retromer can be employed as efficient tools to finely dissect the role of new and established interactors in a quantitative way using multiple methodological approaches (Steinberg et al., 2013). By utilizing numerous structurally distinct cargos, a major conclusion of the present study is the underlying heterogeneity in the molecular details of SNX27–retromer-dependent endosome-to-plasma-membrane recycling.

Interestingly, of the new interactors, or any of the established interactors, none appeared to be fully essential for core SNX27–retromer function, as their efficient knockdown resulted in only partial or cargo-specific phenotypes. The exception was ANKRD50, a 220-kDa protein that contains 19 ankyrin repeats flanked by two termini with no homology to known functional domains. Nothing is known about this protein but our data has established that it is essential for SNX27–retromer function, as its knockdown strikingly phenocopies the loss of this sorting complex across multiple cargos. Precisely what role ANKRD50 plays in SNX27–retromer function remains to be described, and experiments are currently underway to elucidate this.

Our analysis has also established that the WASH complex is not essential for the recycling of all SNX27–retromer cargos. Its suppression does not affect MCT1 sorting and has only minor effects on the sorting of CD97 and TRAILR1. Thus, not all SNX27–retromer cargos appear to require actin polymerization by the WASH complex for efficient sorting. Moreover, it is interesting to note that VARP suppression affected all of those cargos (MCT1, CD97 and TRAILR1) that were not affected by the suppression of WASH complex function. VARP therefore appears to be essential for the sorting of these non-WASH-dependent cargos. The molecular reason(s) behind the differences in the itineraries of these cargos certainly warrants further investigation.

For several reasons, we focused our study on the newly identified retromer interactor VARP. VARP has emerged as a regulator of late endosomal dynamics through its effect on VAMP7 (Schäfer et al., 2012). It plays an established role in melanosome biogenesis (Tamura et al., 2009) and acts as a GEF for endosome-associated Rab21 (Zhang et al., 2006). Perhaps most striking from our analysis of VARP was the apparent cargo specificity in SNX27–retromer-mediated sorting. Whereas knockdown of VARP had only a minor effect on GLUT1 trafficking, strong effects were observed on the SNX27–retromer-dependent sorting of MCT1 and CD97, suggesting that VARP is essential for the transport of a subset of SNX27–retromer cargo. Importantly, in describing a role for retromer in VARP-mediated transport of tyrosinase and Tyrp1, we provide a functional link between retromer and a transport pathway that requires the Rab32/Rab38 and VAMP7 binding activities of VARP.

Besides this specific role in melanosome biogenesis, the identification of VARP as a retromer accessory protein provides a further molecular link bridging retromer with the timing of Rab GTPase switches. Direct binding of VPS29 to the proposed Rab7 GTPase-activating protein TBC1D5 has been implicated in the control of retromer targeting to the late endosome through modifying the availability of Rab7–GTP for binding to VPS35 (Seaman et al., 2009). TBC1D5 also binds to the ubiquitin-like autophagy protein ATG8, an interaction that, by titrating out binding to VPS29, allows TBC1D5 to switch late endosome trafficking itineraries towards autophagosome biogenesis upon starvation (Popovic et al., 2012; Popovic and Dikic, 2014). The diverse and functionally distinct roles of VARP in binding to active forms of late-endosome-associated Rab32 and Rab38 while activating Rab21 on early endosomes (Simpson et al., 2004) suggest that the retromer–VARP association might constitute a point of regulation in switching between early and late endosome transport events.

Finally, the described role of VARP in controlling the fusogenic state of VAMP7 and limiting late-endosome–lysosome fusion (Schäfer et al., 2012), is of interest in the context of a cargo-sorting complex (i.e. retromer) antagonizing and hence controlling the timing of this terminal step in the endosome maturation pathway. Given the decrease in endogenous VARP levels upon retromer suppression in HeLa cells, it is tempting to postulate that by regulating the stability and availability of VARP on endosomes, retromer, in concert with Rab32 and Rab38, might regulate the role of VARP as a kinetic inhibitor of VAMP7-mediated late-endosome–lysosome fusion (Schäfer et al., 2012). Alongside the proposed function of yeast retromer in antagonizing Ypt7-regulated endosome tethering and fusion with the vacuole (Balderhaar et al., 2010; Liu et al., 2012), multiple mechanisms appear to have evolved through which retromer might act as a check-point to prevent premature late-endosome–lysosome fusion. One can speculate that this constitutes a multi-layered ‘proof-reading’ mechanism to ensure that all relevant retromer cargos have been sorted for retrieval before fusion with the lysosome proceeds.

During the review of our paper, David Owen and colleagues also identified the interaction between retromer and VARP (Hesketh et al., 2014). Importantly, they establish that VARP directly binds to VPS29, and that VARP can simultaneously bind to VPS29 and VAMP7. They propose that the interaction with retromer is necessary for the endosome association of VARP. Moreover, through an imaging-based analysis evidence is presented that VARP, alongside VAMP7, is required for retromer-mediated endosome-to-plasma-membrane recycling of GLUT1, a conclusion inconsistent with the relatively minor effect that we observe upon VARP suppression using our biochemical characterization of this pathway. That aside, taken together, these two independent studies define that one functional role of the VARP–retromer interaction is in endosome-to-plasma-membrane recycling. Finally, like our own conclusion, Owen and colleagues propose that, through its ability to recruit VARP, retromer might control endosome–lysosome fusion (Hesketh et al., 2014).

## MATERIALS AND METHODS

### Antibodies

Antibodies used in the study were against the following proteins; SNX1 (monoclonal; clone 51/SNX1; BD Biosciences, 611482), WASH1 and FAM21 (rabbit polyclonal; Gomez and Billadeau, 2009), strumpellin (rabbit polyclonal; Santa Cruz, 87442), VPS26 (rabbit polyclonal; Epitomics, S1181), VPS35 (Abcam, 97545), GFP (mouse monoclonal; clones 7.1/13.1; Roche, 11814460001), MCT1 (rabbit polyclonal; a gift from Andrew Halestrap, University of Bristol, UK), GLUT1 (rabbit polyclonal; Abcam, 15309), TRAILR1 (mouse monoclonal; clone DR-4-02; Aviva, OASA01719), Itgβ1 (rabbit monoclonal; clone EO1042Y; Abcam, 52971), CD97 (mouse monoclonal; clone VIM3B; Biolegend, 336302), TfnR (mouse monoclonal; clone H68.4, Invitrogen, 13-6890), LAMP1 (rabbit polyclonal; Abcam, 24170), Myc-tag (mouse monoclonal; clone 9E10; AbD Serotec), Myc-tag (sheep polyclonal; made in-house for University of Bristol, UK), RME-8 (rabbit polyclonal; a gift from Peter McPherson, McGill University, Montreal, Canada), VARP (rabbit polyclonal; Abcam, ab108216), ANKRD50 (rabbit polyclonal; Abcam, ab108219), SDCCAG3 (rabbit polyclonal; Proteintech, 15969-1-AP), tyrosinase (goat polyclonal; Santa Cruz, M-19), Tyrp1 (Ta99; mouse monoclonal; Santa Cruz), β-actin (mouse monoclonal; Applied Biological Materials) and VPS35 (goat; Abcam, 10099). Rabbit polyclonal anti-tyrosinase and anti-Tyrp1 antibodies were prepared as described previously (Yatsu et al., 2013).

### SILAC interactome analysis

All SILAC reagents were sourced from Thermo Fisher, except for dialyzed FBS, which was from Sigma. Human RPE1 cells were grown in the SILAC DMEM for at least six passages to achieve full labeling (Steinberg et al., 2012; Steinberg et al., 2013). GFP–VPS35 and GFP were lentivirally transduced before the labeling. Cells were lysed in precipitation buffer (50 mM Tris-HCl, 0.5% NP40, Roche Protease Inhibitor Cocktail), and GFP was precipitated with GFP-trap beads (Chromotek) for 30 min. Samples were separated on Nupage 4–12% precast gels (Invitrogen) and subjected to LC-MS/MS analysis on an Orbitrap Velos (Thermo) mass spectrometer as described previously (Steinberg et al., 2012; Steinberg et al., 2013).

### Computational and statistical analysis

Functional gene annotation for the set of VPS35-interacting proteins was performed using the Database for Annotation, Visualization and Integrated Discovery (DAVID, version 6.7). The Cytoscape plug-in Enrichment Map was used to display overlap between gene ontology terms within Cytoscape (version 2.8.2). Node size was mapped to the number of genes within a Gene Ontology category and node color was mapped to the *P*-value for enrichment of a given category. For network analysis, a network interaction map was built using STRING. The network was visualized in Cytoscape.

### Cell culture, transfection, immunofluorescence and western blot analysis

HeLa and RPE1 cells were maintained in DMEM (Gibco-Invitrogen) plus 10% (v/v) fetal calf serum (Sigma-Aldrich) and penicillin-streptomycin (PAA). For immunofluorescence analysis, plasmids containing the stated constructs were transfected using Lipofectamine LTX reagent (Invitrogen). At 48 h after transfection, cells were fixed in 0.1 M phosphate buffer containing 4% (w/v) paraformaldehyde for 10 min on ice and permeabilized with 0.1% (v/v) Triton X-100 for 5 min. Thereafter, cells were incubated with 0.5% (w/v) bovine serum albumin for 30 min, followed by incubation with the indicated primary antibodies and subsequent incubation with secondary antibodies (Molecular Probes). For nuclear staining, DAPI was used. Images were recorded on a Leica SPE or a Leica AOBS-SP2 confocal microscope. Following acquisition, images were analyzed with the Volocity software package (Perkin Elmer); to filter noise, thresholds were applied uniformly across conditions. After setting of the thresholds, the percentage of colocalization and the Pearson's correlation between the respective channels were quantified with the colocalization tool of the Volocity software. Each colocalization analysis was based on the quantification of ≥150 images. Statistical significance was determined by using an unpaired Student's *t*-test. For immunoprecipitation experiments, the respective cell lines stably transduced with the desired GFP-tagged constructs were lysed in Tris-based immunoprecipitation buffer (50 mM Tris-HCl, 0.5% NP40 and Roche Protease inhibitor cocktail), and GFP was precipitated with GFP-trap beads (Chromotek). Western blots were performed using standard procedures. Detection was carried out on a Li-Cor Odyssey Infrared scanning system using fluorescently labeled secondary antibodies. Melan-a cell culture, siRNA transfection, immunostaining of melanogenic enzymes (tyrosinase and Tyrp1) and bafilomycin treatment were performed as described previously (Yatsu et al., 2013).

### siRNA

For VPS35 suppression, the ON-TARGETplus human SMARTpool (Dharmacon) was used, except when suppressed in cells stably expressing a GFP–VPS35 siRNA-resistant construct, in which case only oligo 3 from the SMARTpool was used. RME-8, ANKRD50 and FAM21 were suppressed using ON-TARGETplus human SMARTpools (Dharmacon). VARP was suppressed using the oligo 5′-AAGAUGUGUCACCCUCUCUGCTT-3′. For GLUT1 immunofluorescence localization experiments, SDCCAG3 was suppressed using the oligo 5′-AAUUCUAAGCUGAGAAGAATT-3′, and for examination of surface levels of GLUT1 and MCT1 a second oligo was also used (5′-UACGACGCACUGAAAGAUGAATT-3′). Mouse VPS35 was suppressed using the following oligos (Nippon Gene; Toyama, Japan): 5′-GGUGUAAAUGUGGAACGUU-3′ (site 1) and 5′-CCUGUAGAAGACCCUGAU C-3′ (site 2).

### Quantification of the surface levels of GLUT1 and MCT1

For the quantification of protein surface levels, cells were surface biotinylated with a commercially available kit (Pierce/Thermo) according to the manufacturer's instructions at 72 h post transfection. Cells were lysed in PBS with 2% Triton X-100, lysates were pooled and streptavidin–agarose (GE-Healthcare) was used to capture biotinylated membrane proteins. After capture, biotinylated proteins were washed extensively in 1.2 M NaCl containing PBS with 1% Triton X-100 to remove cytoskeletal proteins and contaminants from the transmembrane proteins, followed by elution of the proteins by boiling in dithiothreitol-containing sample buffer.

### Flow cytometry and antibody-uptake assays

Flow cytometric detection of TRAILR1 and CD97 was performed as described previously (Steinberg et al., 2012). Antibody-uptake assays were performed as described previously (Steinberg et al., 2013).

### Degradation assays

To measure the degradation of surface proteins, HeLa cells were transfected with the requisite siRNA. At 24 h post transfection, before the effects of the siRNA were fully established, surface proteins were biotinylated with sulpho-NHS-biotin (Pierce/Thermo). Cells were lysed at 0, 12 and 24 h after biotinylation (24, 36 and 48 h post transfection of siRNA), and biotinylated proteins were captured with streptavidin–agarose (GE-Healthcare) and detected by quantitative western blotting on an Odyssey scanner. Degradation was quantified as the fluorescence signal remaining after 12 and 24 h as a percentage of the signal intensity at *t* = 0.

### VPS35 constructs and mutagenesis

VPS35 was subcloned into pXLG3. The shRNA viral construct against the 3′UTR of VPS35 was purchased from Sigma (TRCN0000337019). siRNA-resistant VPS35 was generated by introducing 6 silent base mis-matches (T1314A, T1317C, G1320A, C1321T, T1323A, T1326C) into the open reading frame, conferring resistance to siRNA VPS35-3 described above.

### Statistical analysis

All quantified western blot and confocal colocalization data are shown as the mean of the indicated number of independent experiments. The raw data from the quantitative western blotting were first normalized to percentage of control (comparative western blots) or to percentage of *t* = 0 (degradation assays) for each condition. The mean and standard error of the mean over the indicated number of independent experiments were calculated, followed by performing an unpaired Student's *t*-test to determine statistical significance. Colocalization data from at least three independent experiments was averaged across individual experiments and standard deviation was calculated across the three experiments. An unpaired Student's *t*-test was then used to analyze statistical significance. For all statistical tests, a *P*-value of <0.05 was considered significant and is indicated by an asterisk.

## Supplementary Material

Supplementary Material
