# Supplementary material for: Identification of molecular heterogeneity in SNX27–retromer-mediated endosome-to-plasma-membrane recycling
Source: J Cell Sci. 2014 Nov 15;127(22):4940–53. doi: 10.1242/jcs.156299 (PMC4231307; doi:10.1242/jcs.156299)
Supplement: Supplementary Material [file supp_127_22_4940__index.html]

Identification of molecular heterogeneity in SNX27–retromer-mediated endosome-to-plasma-membrane recycling — Supplementary Material 

# Identification of molecular heterogeneity in SNX27–retromer-mediated endosome-to-plasma-membrane recycling

## JCS156299 Supplementary Material

**Files in this Data Supplement:**

- **Supplementary Material**
