# Supplementary material for: Identification of molecular heterogeneity in SNX27–retromer-mediated endosome-to-plasma-membrane recycling
Source: J Cell Sci. 2014 Nov 15;127(22):4940–53. doi: 10.1242/jcs.156299 (PMC4231307; doi:10.1242/jcs.156299)
Supplement: Supplementary Material [file supp_127.22.4940_JCS156299.pdf]

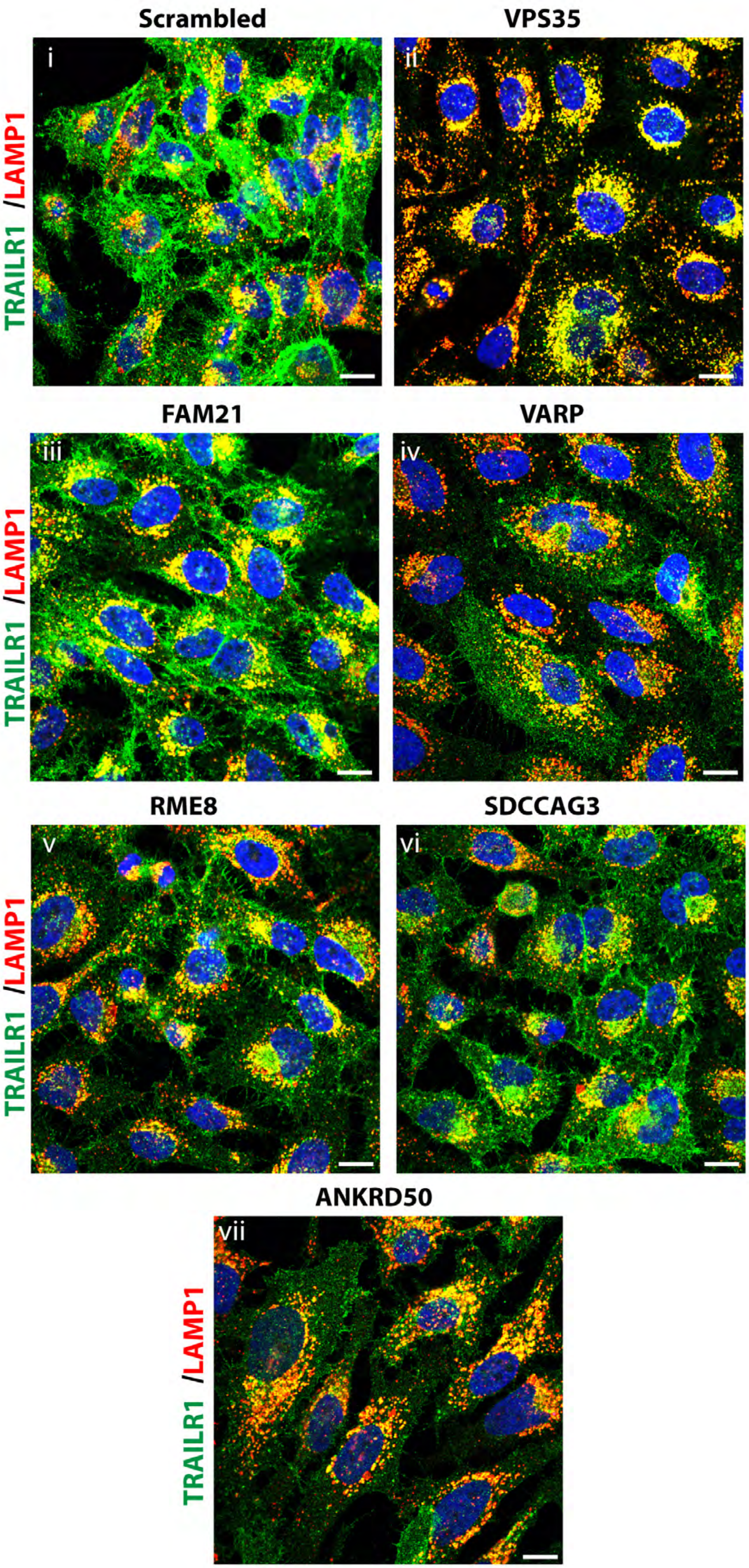
Scrambled
VPS35
FAM21
VARP
RME8
SDCCAG3
ANKRD50
i
ii
iii
iv
v
vi
vii
TRAILR1 /LAMP1
TRAILR1 /LAMP1
TRAILR1 /LAMP1
TRAILR1 /LAMP1

Fig. S1. VPS35 and ANKRD50 suppression leads to an increase in the lysosomal accumulation of TRAILR1. HeLa cells were transfected with siRNA against the indicated targets and 72 hours later incubated with an anti-TRAILR1 antibody targeting an exofacial epitope of this receptor. After a 1 hour incubation at 37°C, to allow uptake and recycling of the tagged TRAILR1, cells were fixed and the localisation of the TRAILR1 was determined through detection of the anti-TRAILR1 antibody. Co-staining for endogenous LAMP1 allow visualisation of the spatial relationship of TRAILR1 and late endosome/lysosomes. Scale bars: 10 μm.

Table S1. SILAC quantified interactome GFP-VPS35 vs. GFP from human RPE1 cells

Download Table S1

Table S2. SILAC quantified and filtered interactome GFP-VPS35 vs. GFP from human RPE1 cells

Download Table S2
